# Correlation between reduction of superior interventricular groove epicardial fat thickness and improvement of insulin resistance after weight loss in obese men

**DOI:** 10.1186/1758-5996-6-115

**Published:** 2014-10-29

**Authors:** Kae-Woei Liang, I-Chen Tsai, Wen-Jane Lee, Shih-Yi Lin, Wen-Lieng Lee, I-Te Lee, Chia-Po Fu, Jun-Sing Wang, Wayne H-H Sheu

**Affiliations:** Cardiovascular Center, Taichung Veterans General Hospital, No.1650 Section 4, Taiwan Boulevard, Taichung, 40705 Taiwan; Cardiovascular Research Center, Department of Radiology and Department of Medicine, National Yang Ming University School of Medicine, Taipei, Taiwan; Department of Medicine, Chung Shan Medical University, Taichung, Taiwan; Department of Medicine, China Medical University, Taichung, Taiwan; Department of Radiology, Taichung Veterans General Hospital, Taichung, Taiwan; Department of Medical Imaging, Show Chwan Memorial Hospital, Changhua, Taiwan; Department of Medical Imaging, Chang Bing Show Chwan Memorial Hospital, Changhua, Taiwan; Department of Medical Research, Taichung Veterans General Hospital, Taichung, Taiwan; Tung-Hai University, Taichung, Taiwan; Division of Endocrinology and Metabolism, Department of Medicine, Taichung Veterans General Hospital, Taichung, Taiwan; Institute of Biomedical Sciences, National Chung Hsing University, Taichung, Taiwan; Taichung Veterans General Hospital, No.1650 Section 4, Taiwan Boulevard, Taichung, 40705 Taiwan

**Keywords:** Epicardial adipose tissue, Insulin resistance, Leptin, Matsuda index, Metabolic syndrome, Obesity

## Abstract

**Background:**

It has been recognized that reduction of abdominal visceral fat and subcutaneous fat are associated with improvement in insulin-resistance (IR) after weight loss. However, few studies have investigated the correlation of reduction in epicardial adipose tissue (EAT) with improvement of IR index after weight loss in obese non-diabetic men with metabolic syndrome (MetS).

**Methods and results:**

We prospectively enrolled 32 non-diabetic men with MetS for a 3-month weight reduction program mainly by diet control and exercise. Magnetic resonance imaging (MRI) examinations were used to measure EAT, subcutaneous fat, and abdominal visceral fat. Anthropometric parameters, oral glucose tolerance test (OGTT), and serum adipokines were assessed before and after the weight loss program. After a 3-month weight loss program, 27 obese MetS men had significant weight loss >5% (97 ± 14 to 87 ± 14 kg, with a 10.7 % decrease, p < 0.001). Multivariate analysis revealed that the decrement ratio of superior interventricular groove (SIVG) EAT thickness (*r* = 0.322, p = 0.044) and serum leptin (*r* = 0.626, p < 0.001) significantly correlated with the percentage improvements of fasting HOMA-IR index. Furthermore, the decrement ratio of SIVG EAT thickness (*r* = −0.370, p = 0.017) and decrement ratio of subcutaneous fat area (*r* = −0.673, p = 0.006) were significantly correlated with improvement of OGTT-derived Matsuda insulin-sensitivity index.

**Conclusions:**

The decrement ratio of SIVG EAT correlated with improvement of both HOMA-IR and OGTT-derived Matsuda insulin-sensitivity indexes after weight loss in obese non-diabetic men with MetS.

**Clinical trial registration:**

(Multi-faceted Evaluations Following Weight Reduction in Subjects with Metabolic Syndrome NCT 01065753 on Feb 8, 2010).

## Introduction

The core pathogenesis of metabolic syndrome (MetS) is related to obesity and insulin resistance (IR) [1]. MetS is associated with a higher incidence of cardiovascular diseases and events, and increased cardiovascular mortality [2–5]. We and others have demonstrated that victims of MetS manifest a pro-thrombotic and pro-inflammatory state [6–8]. Even in subjects without type 2 diabetes mellitus, MetS alone still carries a 1.6-fold increased risk of myocardial infarction and a 1.8-fold increased risk of cardiovascular mortality [5].

Epicardial adipose tissue (EAT) is a part of visceral fat deposited around the heart between the pericardium and myocardium [9]. The distribution of EAT is asymmetric and mostly localizes at perivascular, interventricular, and atrioventricular grooves [10, 11]. Animal study showed that the rate of insulin induced lipogenesis was significantly higher in EAT compared with other fat depots [12]. In addition to storing lipids, it also produces inflammatory cytokines and adipokines [9, 13, 14]. Measurements of EAT by computed tomography (CT) or echocardiogram have been used for predicting components of MetS or coronary atherosclerosis severity [15–18]. Some studies have addressed the possible mechanism of EAT in coronary atherosclerosis, such as an increase of local EAT leptin [19] and inflammatory cytokine secretion [13], or a decrease of adiponectin production [14], which might directly be diffused into adjacent coronary circulation and influence atherosclerosis [10]. In fact, it has been shown that leptin expression was higher in EAT obtained from subjects with MetS or coronary heart disease [19, 20]. However, whether total EAT volume [16, 21], average EAT thickness [22] or regional EAT thickness [15, 18] better predicts coronary atherosclerosis or MetS is still under debate and whether there is a regional specific EAT difference in atherogenesis is still inconclusive [10]. Recently, we reported that differences in right atrio-ventricular groove EAT thickness can help differentiate the inflammatory status of obese non-diabetic men with MetS [23].

Weight loss improves IR, decreases circulating leptin and increases adiponectin in obesity or MetS [24–26]. There have been inconsistent reports whether reduction of abdominal visceral fat or subcutaneous fat is better correlated with the improvement of IR after weight loss [27–31]. In addition, few studies have investigated the correlations of reductions of regional EAT with improvement of IR after weight loss in obesity or MetS. Moreover, alterations of circulating adipokine change and improvement of IR following weight loss in obese non-diabetic men with MetS was largely unexplored. The aims of this study were to elucidate the correlations of regional EAT thickness changes by magnetic resonance imaging (MRI) with improvement of IR and changes in adipokines after weight loss in obese non-diabetic men with MetS.

## Methods

### Study subjects

**Figure 1:**
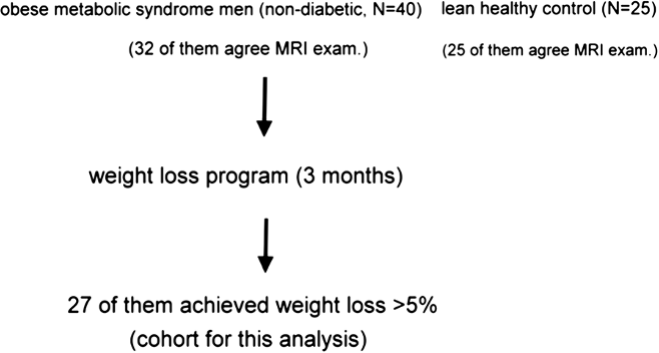
**Study enrollment and protocol.** MRI: magnetic resonance imaging.

We prospectively enrolled 40 men in total, who were diagnosed with MetS using the ATP-III criteria, from outpatient clinics at Taichung Veterans General Hospital (Taichung, Taiwan) in April to September 2008 (Figure 1). Parts of the results have been published previously [23, 25, 26]. In brief, the study enrollment criteria included central obesity, which was defined as waist circumference ≥90 cm, in addition to at least two other ATP-III MetS criteria [32]. Patients with diabetes mellitus requiring oral anti-diabetic agents or insulin shots were excluded from the study. The patients undertook a 3-month weight reduction program. The dietitian instructed the study subjects to consume a 1200 Kcal diet per day comprising 55% carbohydrate, 30% fat, and 15% protein. Patients were educated how to estimate the calories and nutritional components per unit amount of food [25, 33, 34]. They all received an instruction brochure, which stated all the nutritional facts and calories per unit amount of food for instant check. The patients were asked to keep a very detailed record of each meal. A dietitian would estimate the calories based on the record in order to keep the daily requirement of 1200Kcal per day. In addition, the dietitian closely followed them with phone-call consultations. We encouraged peer competition for weight loss and showed weight and waist circumference changes in bar graphs at each gathering, which was held once every week in the first month, then biweekly in the 2nd and 3rd month. The patients were encouraged to have at least two to three light physical exercise periods per week. We provided a 60-minute fitness program with a physical trainer at the biweekly gatherings. The study subjects underwent anthropometric data check and blood sampling again at the 3^rd^, 12^th^ and 18^th^ month after the beginning of weight loss program and then every 24 months thereafter if they are compliant with our recalls. Thirty-two of the obese non-diabetic men with MetS agreed undergoing MRI examination for measurement of subcutaneous fat, abdominal visceral fat, and EAT before and after weight loss (Figure 1). All subjects provided written informed consent. The authors confirm that all ongoing and related trials for this study/intervention are registered. The study protocol was approved by the Human Research Ethics Review Committee of Taichung Veterans General Hospital (Taichung, Taiwan). The protocol and case enrollment were conducted after getting the ethics committee approval; however, the registration at http://www.clinicaltrials.gov (Multi-faceted Evaluations Following Weight Reduction in Subjects with Metabolic Syndrome NCT 01065753) was delayed till Feb 8, 2010 because clinical trial registration was not compulsory in our country back then.

### Biochemical analysis

All obese MetS subjects underwent blood tests and oral glucose tolerance test (OGTT) after an overnight fast to exclude those with unknown diabetes. After a fasting blood sample was collected, glucose load of 75 g was ingested over 5 minutes. Blood samples were collected at 30 minutes, 60 minutes, 90 minutes, and 120 minutes after the test load. Blood glucose and insulin concentrations were measured in each sample. Serum insulin was determined by a commercially available assay kit (IMMULITE, I-2000, EURO/Diagnostic Products Corporation, Gwynedd, UK). The inter- and intra-assay coefficients of variation for insulin (range 10.7 to 439 μU/ml) were 4.3% and 5.4%, respectively. Insulin-resistance was estimated using the homeostasis model assessment of IR (HOMA-IR), defined as fasting glucose mg/dl × fasting insulin μU/mL/405 [35, 36]. OGTT-derived Matsuda insulin-sensitivity index (ISI) was defined as 10,000/sqrt [fasting glucose (mM) × fasting insulin (μU/mL) × mean glucose during OGTT (mM) × mean insulin during OGTT (μU/mL)] [37]. OGTT glucose and insulin areas under the curve (AUC) were defined as areas under the curve for glucose and insulin vs. time during the OGTT, respectively [8]. Serum hs-CRP was determined by particle-enhanced immunoturbidimetry (Latex microparticles sensitized with duck anti-CRP IgY kit, provided by Good Biotech Corp., Taichung, Taiwan). The intra- and inter-assay coefficients of variance were 1.4% and 1.42%, respectively [8]. Serum monocyte chemotactic protein (MCP)-1 was determined by enzyme-linked immunosorbent assays (R&D Systems, Inc., Minneapolis, MN, USA) [38, 39]. The intra- and inter-assay coefficients of variation for MCP-1 were 5.80% and 5.70%, respectively, with a minimum detectable concentration of <5.0 pg/mL.

### Measurement of circulating adipokines

Serum adiponectin, leptin, and plasma plasminogen activator inhibitor (PAI)-1 concentrations were determined by enzyme-linked immunosorbent assays (R&D Systems, Inc., Minneapolis, MN, USA). The intra-assay and inter-assay coefficients of variation of PAI-1 were 6.73% and 7.33%, respectively, with a minimum detectable concentration of 0.014-0.142 ng/mL. The intra- and inter-assay coefficients of variation for adiponectin were 3.53% and 6.50%, respectively, with a minimum detectable concentration of 0.079-0.891 ng/mL. The intra- and inter-assay coefficients of variation for leptin were 3.17% and 4.37%, respectively, with a minimum detectable concentration of <7.8 pg/mL.

### MRI examination for subcutaneous fat, abdominal visceral fat, and EAT

Subcutaneous fat and abdominal visceral fat areas were quantified using MRI. Data were acquired using a 1.5 T whole body Siemens Sonata system (Siemens Medical Systems, Erlangen, Germany) using the body coil. For the quantification of abdominal fat, one axial slice of the abdomen was obtained at the level of umbilicus. The scan parameters used were TR =209 ms, TE =4.1 ms, slice thickness =10 mm, and flip angle =70°. The field of view was 400 mm with an in-plane image resolution of 2.4 mm × 1.6 mm [40]. Areas of abdominal visceral fat and subcutaneous fat (millimeters squared) were measured on the computer screen using a track-ball [41]. All measurements were analyzed by a single radiologist who was blinded to the metabolic status of the study subjects.

For EAT measurement, electrocardiogram-gated cine images were acquired with a dedicated 4-element, phased array cardiac coil, using a segmented steady-state free precession [fast imaging with steady-state precession (TrueFISP)] sequence (time to echo/time of repetition 1.6/3.2 ms, temporal resolution 35 ms, in-plane spatial resolution 1.4*1.8 mm, slice thickness 5 mm, inter-slice gap 5 mm). The measurements were done in end-diastolic phase on horizontal long-axis plane and basal short-axis plane (at the level of the end of papillary muscles). Fat thickness in right atrioventricular groove, left atrioventricular groove, and anterior interventricular groove were measured on horizontal long-axis plane; fat thickness in superior interventricular groove, inferior interventricular groove and right ventricular free wall were measured on basal short-axis plane, as described in our previous studies and by other investigators [15, 23, 26, 42].

### Statistical analysis

Continuous variables are expressed as mean ± S.D. Differences in continuous variables before and after weight loss in obese MetS subjects were compared by the paired Student’s t test. Univariate correlation analysis between percentage changes of HOMA-IR or ISI-Matsuda after weight loss vs. percentage changes of obesity-associated inflammatory markers, adipokines, and MRI-measured fat index in obese MetS were analyzed using Pearson correlation analysis. Multivariate linear regression analysis was performed to investigate the independent variables correlated with percentage changes of HOMA-IR or ISI-Matsuda after weight loss. The SPSS 17.0 statistical software package (SPSS, Inc., Chicago, IL, USA) was used for all calculations. A two-tailed p value less than 0.05 was considered statistically significant.

## Results

### Changes in anthropometric, serum or plasma biochemical data, and adipokines after weight loss

After the 3-month weight reduction program, twenty-seven of the obese non-diabetic men with MetS achieved weight loss of more than 5% (97 ± 14 to 87 ± 14 kg, with an average decrease of 10.7%, p < 0.001) (Figure 1). The fasting HOMA-IR, OGTT-derived Matsuda ISI, and OGTT glucose or insulin AUC changed significantly after weight loss (Table 1). Lipid profiles improved and inflammatory markers such as MCP-1 declined after weight loss (Table 1). In terms of circulating adipokines, the obese non-diabetic men had significantly lower values of PAI-1 after weight loss. There were non-significant changes of adiponectin and leptin levels after weight loss (Table 1).

**Table 1:** **Changes of anthropometric, serum or plasma biochemical data and adipokines in obese non-diabetic men with metabolic syndrome with weight loss >5% (N = 27) by diet and exercise program**

	Before (N = 27)	After (N = 27)	*Percentage of changes	***P value***
Body weight (kg)	97 ± 14	87 ± 14	−10.7%	<0.001
BMI (kg/m^2^)	33.5 ± 4.1	29.9 ± 4.2	−10.6%	<0.001
Fasting glucose (mg/dl)	103 ± 15	97 ± 10	−5.7%	0.002
Fasting insulin (μIU/ml)	18.6 ± 12.1	13.0 ± 7.3	−25.8%	0.005
HOMA-IR (mg/dl × μIU/ml)	4.8 ± 3.2	3.1 ± 1.7	−29.5%	0.003
OGTT glucose AUC (mg/dl × h)	367 ± 79	324 ± 55	−9.8%	<0.001
OGTT insulin AUC (μIU/ml × h)	200 ± 99	148 ± 75	−21.1%	0.002
OGTT derived insulin-sensitivity index, Matsuda (mM × μIU/ml)^−1^	40.6 ± 16.6	64.6 ± 31.2	69.7%	<0.001
Total cholesterol (mg/dl)	204 ± 51	187 ± 35	−7.2%	0.011
HDL-C (mg/dl)	40 ± 9	44 ± 10	11.2%	0.021
Triglyceride (mg/dl)	295 ± 515	174 ± 294	−36.6%	0.012
MCP-1 (pg/ml)	277 ± 88	215 ± 66	−19.5%	<0.001
Hs-CRP (mg/dL)	0.29 ± 0.41	0.14 ± 0.11	−19.3%	0.061
**Adipokines**				
Adiponectin (ng/ml)	3197 ± 1803	3377 ± 2140	35.5%	0.431
Leptin (pg/ml)	16034 ± 9932	11673 ± 11362	−21.2%	0.067
PAI-1 (ng/ml)	18.4 ± 8.2	9.7 ± 4.9	−37.0%	<0.001

BMI (kg/m^2^): body mass index = body weight (kg)/height^2^ (m).

HDL-C: high-density-lipoprotein cholesterol.

HOMA-IR: homeostasis model assessment (HOMA) index of insulin resistance = (fasting glucose mg/dl × fasting insulin μU/mL)/405.

MCP-1: monocyte chemotactic protein-1.

MetS: metabolic syndrome.

OGTT glucose AUC: oral glucose tolerance test, area under the curve for glucose.

OGTT insulin AUC: oral glucose tolerance test, area under the curve for insulin.

OGTT insulin-sensitivity index (Matsuda): 10,000⁄sqrt (FBG × FPI × G × I) where FPG is fasting glucose (mM), FPI is fasting insulin (μIU/ml), G is mean glucose (mM), I is mean insulin (μIU/ml) during the OGTT.

PAI-1: plasminogen activator inhibitor-1.

*Percentage of changes = (after - before)/before weight loss, mean value.

### Changes of MRI measured subcutaneous fat, abdominal visceral fat, and regional thickness of EAT in obese non-diabetic men with MetS after weight loss

Significant reductions of subcutaneous fat and abdominal visceral fat areas as well as decreases of regional EAT thickness were detected in the 27 obese men who achieved significant weight loss (all p < 0.001) (Table 2).

**Table 2:** **Changes of MRI measured subcutaneous fat, abdominal visceral fat, and regional thickness of epicardial adipose tissue in obese non-diabetic men with metabolic syndrome with weight loss >5% (N = 27)**

	Before (N = 27)	After (N = 27)	*Percentage of changes	***P value***
Waist (cm)	122.6 ± 10.0	112.8 ± 11.4	−8%	<0.001
Subcutaneous fat area (mm^2^)	34825 ± 10493	25005 ± 9146	−28%	<0.001
Abdominal visceral fat area (mm^2^)	47469 ± 6344	41413 ± 7751	−13%	<0.001
Epicardial adipose tissue thickness (mm)				
*Basal short axis plane*				
SIVG	10.0 ± 3.4	6.4 ± 2.2	−34%	<0.001
IIVG	10.5 ± 4.7	6.3 ± 2.3	−31%	<0.001
RVFW	6.8 ± 1.9	4.4 ± 1.3	−33%	<0.001
*Horizontal long axis plane*				
RAVG	16.7 ± 3.5	13.2 ± 2.3	−18%	<0.001
LAVG	15.5 ± 3.8	10.3 ± 3.1	−32%	<0.001
AIVG	9.2 ± 2.4	6.6 ± 1.5	−24%	<0.001

AIVG: anterior interventricular groove; IIVG: inferior interventricular groove; LAVG: left atrioventricular groove; RAVG: right atrioventricular groove; RVFW: right ventricular free wall; SIVG: superior interventricular groove.

*Percentage of changes = (after - before)/before weight loss, mean value.

### Univariate correlation analysis of percentage changes in HOMA-IR index with obesity-associated inflammatory markers, adipokines, and MRI-measured fat index after weight loss

The percentage improvements in HOMA- IR index after weight loss were significantly correlated with the MRI-measured decrement ratio of superior interventricular groove (SIVG) EAT thickness (*r* =0.408, p = 0.035), but not with the subcutaneous fat or abdominal visceral fat changes (Table 3). Moreover, the percentage improvement of HOMA-IR index was also significantly correlated with decrement ratio of secretory leptin after weight loss (*r* = 0.685, p < 0.001) (Table 3).

**Table 3:** Univariate correlation analysis of *percentage changes of fasting HOMA-IR and OGTT-derived insulin-sensitivity index (Matsuda) with *percentage changes of obesity-associated inflammatory markers, adipokines and MRI measured fat index (study subjects: 27 obese non-diabetic men with metabolic syndrome, with weight loss >5%)

Factors (*percentage changes)	HOMA-IR		ISI-Matsuda	
	Correlation coefficients (***r***)	***p***	Correlation coefficients (***r***)	***p***
**BMI**	0.201	0.314	−0.485	0.010
**MR measured fat index**				
waist	−0.014	0.944	−0.434	0.024
Subcutaneous fat area	0.216	0.280	−0.642	<0.001
Abdominal visceral fat area	0.094	0.641	−0.369	0.058
Epicardial adipose tissue thickness				
*Basal short axis plane*				
SIVG	0.408	0.035	−0.501	0.008
IIVG	0.259	0.193	−0.359	0.066
RVFW	0.342	0.080	−0.201	0.314
*Horizontal long axis plane*				
RAVG	−0.049	0.808	−0.213	0.287
LAVG	−0.064	0.753	−0191	0.340
AIVG	0.018	0.929	−0.198	0.321
HDL-C	−0.001	0.995	0.195	0.329
Triglyceride	0.037	0.856	−0.234	0.240
MCP-1	−0.164	0.414	0.050	0.803
HsCRP	−0.003	0.989	−0.022	0.911
**Adipokines**				
Adiponectin	0.093	0.643	0.269	0.175
Leptin	0.685	<0.001	−0.439	0.022
PAI	−0.028	0.888	−0.176	0.379

AIVG: anterior interventricular groove; BMI (kg/m^2^): body mass index = body weight (kg)/height^2^ (m); HDL-C: high-density lipoprotein cholesterol; HOMA-IR: homeostasis model assessment of insulin resistance, defined as glucose mg/dl × insulin μU/mL/405; Hs-CRP: high sensitivity C-reactive protein; IIVG: inferior interventricular groove; LAVG: left atrioventricular groove; MCP-1: monocyte chemotactic protein-1; Oral glucose tolerance test (OGTT)-derived insulin-sensitivity index (ISI), Matsuda: 10,000/sqrt (FBG × FPI × G × I), where FPG is fasting glucose (mM), FPI is fasting insulin (μIU/ml), G is mean glucose (mM), I is mean insulin (μIU/ml) during the OGTT; PAI-1: plasminogen activator protein-1; RAVG: right atrioventricular groove; RVFW: right ventricular free wall; SIVG: superior interventricular groove.

*Percentage of changes = (after - before)/before weight loss.

### Univariate correlation analysis of percentage changes in OGTT-derived Matsuda insulin-sensitivity index with obesity-associated inflammatory markers, adipokines, and MRI-measured fat index after weight loss

The percentage improvements of OGTT-derived Matsuda ISI were significantly correlated with MRI-measured decrement ratio of SIVG EAT thickness (*r* = −0.501, p = 0.008) as well as with the subcutaneous fat (*r* = −0.642, p < 0.001), but not with abdominal visceral fat changes after weight loss (Table 3). Moreover, the percentage improvement of OGTT-derived Matsuda ISI was also significantly correlated with decrement ratio of secretory leptin after weight loss (*r* = −0.439, p = 0.022) (Table 3). In a separate analysis, the decrement ratio of SIVG EAT also correlated well with the decrement ratio of OGTT glucose AUC (*r* =0.520, p = 0.005). The correlation between decrement ratio of SIVG EAT and the decrement ratio of OGTT insulin AUC was fair but not reaching statistical significance (*r* =0.327, p = 0.095). Moreover, there was no correlation between absolute difference of MR measured left ventricular ejection fraction vs. HOMA-IR (*r* = −0.035, p = 0.863) or OGTT-derived Matsuda ISI (*r* =0.067, p = 0.742) changes after weight loss.

### Multivariate linear regression analysis of independent variables correlated with percentage changes of HOMA-IR index in obese non-diabetic men with MetS after weight loss

In a multivariate linear regression analysis, the decrement ratio of circulating leptin and SIVG EAT were independent variables correlated with improvements of HOMA-IR index after weight loss, while changes in subcutaneous or abdominal visceral fat were not (Table 4).

**Table 4:** **Multivariate linear regression analysis of variables related with percentage of changes of HOMA-IR index in obese non-diabetic men with metabolic syndrome after weight loss >5% (N = 27)**

Predictor (*percentage of changes)	Standardized coefficient β	***p***
Subcutaneous fat area	0.090	0.609
Abdominal visceral fat area	−0.148	0.408
SIVG EAT thickness	0.322	0.044
Leptin	0.626	<0.001

Dependent variable: percentage changes of HOMA-IR.

EAT: epicardial adipose tissue.

SIVG: superior interventricular groove.

HOMA-IR: homeostasis model assessment (HOMA) of insulin resistance = (fasting glucose mg/dl × fasting insulin μU/mL)/405.

*Percentage of changes = (after - before)/before weight loss.

### Multivariate linear regression analysis of independent variables correlated with percentage changes of Matsuda insulin-sensitivity index in obese non-diabetic men with MetS after weight loss

In a multivariate linear regression analysis, the decrement ratio of SIVG EAT thickness (*r* = −0.370, p = 0.017) and subcutaneous fat area (*r* = −0.673, p = 0.006) were independent variables correlated with the increment ratio of OGTT-derived Matsuda ISI after weight loss (Table 5).

**Table 5:** **Multivariate linear regression analysis of variables related with percentage of changes of OGTT-derived insulin-sensitivity Matsuda index, in obese non-diabetic men with metabolic syndrome with weight loss >5% (N = 27)**

Predictor (*percentage of changes)	Standardized coefficient β	***p***
Body mass index	0.194	0.458
Subcutaneous fat area	−0.673	0.006
Abdominal visceral fat area	0.030	0.877
SIVG EAT thickness	−0.370	0.017
Leptin	−0.269	0.065

Dependent variable: percentage changes of Matsuda index.

EAT: epicardial adipose tissue.

SIVG: superior interventricular groove.

OGTT-derived insulin-sensitivity index (Matsuda): 10,000⁄sqrt (FBG × FPI × G × I) where FPG is fasting glucose (mM), FPI is fasting insulin (μIU/ml), G is mean glucose (mM), I is mean insulin (μIU/ml) during the OGTT.

*Percentage of changes = (after - before)/before weight loss.

## Discussion

The results of this study demonstrated that the decrement ratio of SIVG EAT correlated with improvement of both HOMA-IR and OGTT-derived Matsuda insulin-sensitivity indexes after weight loss in obese non-diabetic men with MetS. In this study, we applied a weight loss intervention to investigate its impact on changes of insulin-resistance in non-diabetic obese men and extended the investigation parameters beyond subcutaneous or abdominal visceral fat to regional EAT changes.

A previous report by Iacobellis et al. [43] showed that EAT thickness measured by echocardiogram over the right ventricle free wall was significantly correlated with IR index in obese subjects. Manco et al. [44] reported that MRI-measured EAT volume was a useful predictor for HOMA-IR in obese children. Our study further corroborated the association of EAT with IR, and revealed a novel finding showing that MRI-measured regional SIVG EAT thickness change was independently associated with improvement of IR after weight loss in obese non-diabetic men with MetS. Moreover, we used multiple IR indexes, including the HOMA-IR and Matsuda ISI to validate the association between SIVG EAT and IR changes.

EAT is metabolically active visceral fat that produces inflammatory cytokines and adipokines, including leptin [13, 14, 19]. Some studies have addressed the possible role of EAT in coronary atherosclerosis, such as an increase of local EAT leptin [19, 20], and inflammatory cytokine secretion [13], or a decrease of adiponectin production [14], which might directly be diffused into adjacent coronary circulation and influence atherosclerosis. Subjects with MetS or coronary heart disease have been reported to have higher leptin expression in EAT [19, 20]. Beyond the local production of leptin in EAT, there was also increased circulating leptin levels in obesity, metabolic syndrome, and IR state [25, 45]. Weight loss decreased circulating leptin and the changes of HOMA-IR related with changes of leptin after weight reduction in obese children [45–47]. Our study further confirmed that the decrement ratio of secretory leptin was independently associated with improvement of HOMA-IR after weight loss in obese non-diabetic men with MetS. In contrast, other adipokines such as, adiponectin or PAI-1 changes did not correlate well with improvement of HOMA-IR.

EAT surrounding the thin-walled coronary veins may exert greater systemic effects by diffusion of its small metabolically active molecules into the circulation [10, 18]. The great cardiac vein traverses the anterior inter-ventricular groove and is embedded by a substantial amount of EAT. Given the amount of fat around and the presence of a larger drainage territories of the anterior wall of the heart into the great cardiac vein, this may partly explain why only changes of EAT thickness in the SIVG was associated with the IR changes after weight loss in obese men with MetS [10, 18].

In our study, the decrement ratio of subcutaneous fat rather than abdominal visceral fat correlated with improvement of Matsuda ISI after weight loss in obese non-diabetic men with MetS. The decrement ratio of abdominal visceral fat was neither correlated with changes of HOMA-IR or ISI-Matusda after weight loss in this study. A previous report by Klein et al. [27] showed that liposuction of subcutaneous fat did not alter the insulin-sensitivity, or circulating hs-CRP or adiponectin in obese women. However, Kremen et al. [48] found that both subcutaneous and EAT were sources of pro-inflammatory cytokines in cardiac surgery patients and may contribute to the development of postoperative insulin resistance. Another report analyzing local fat sample from subjects undergoing coronary bypass surgery showed that EAT produced more inflammatory molecules than subcutaneous adipose tissue [13]. In addition, studies by Grundy et al. also showed that subcutaneous truncal fat played a major role in obesity-related insulin resistance in men, whereas intraperitoneal fat and retroperitoneal fat had a lesser role [30, 31]. Kaess et al. [49] proposed visceral fat vs. subcutaneous fat ratio as a better correlate of cardio-metabolic risk, above and beyond BMI and visceral fat alone. These discrepancies may be caused by different ethnic groups, gender, and the methods employed. Certainly, further studies are required to investigate the changes in subcutaneous or abdominal visceral fat with improvement of IR after weight reduction.

Whether different regional EAT or changes of EAT thickness participate in mechanisms leading to different inflammatory or metabolic risks requires further in vitro and in vivo investigations [18, 19, 23]. This study only observed a useful surrogate EAT parameter (SIVG thickness) change for indicating the improvement of insulin-resistance after weight loss in obese men with MetS. However, the pathogenesis underlying the close correlations between changes of regional EAT and circulating leptin with the improvement of IR after weight loss in obese MetS demands further in vivo animal studies and in vitro cell signaling pathway investigations [19]. This study recruited obese men only and the results might not be applicable to women. The major limitation of the study was small sample size and the results were mainly from clinical data analysis without mechanism investigations. Moreover, this result was a post-hoc subgroup analysis of an original 40 men who participated in a weight loss study by diet and exercise. Among them, only 32 agreed MRI exam. And of whom, a subgroup who had achieved weight loss >5% was entered into analysis for correlation between regional fat change and IR. The potential bias resulted from post hoc subgroup analysis could not be neglected.

In conclusion, the decrement ratio of SIVG EAT thickness was independently associated with improvement of IR index after weight loss in obese non-diabetic men with MetS. The underlying mechanisms linking change in SIVG EAT thickness and improvement of systemic IR require further study.
